# Illness Perception in Adolescent Patients With Anorexia: Does It Play a Role *in socio*-Emotional and Academic Adjustment?

**DOI:** 10.3389/fpsyg.2020.01730

**Published:** 2020-07-21

**Authors:** Yolanda Quiles, Maria José Quiles, Eva León, Javier Manchón

**Affiliations:** Department of Behavioral Sciences and Health, Miguel Hernández University, Elche, Spain

**Keywords:** anorexia nervosa, adolescents, illness perception, socio-emotional adjustment, academic adjustment

## Abstract

People’s beliefs about their illness have been shown to affect their adjustment. The aim of this study was to describe illness perception in adolescent patients with anorexia nervosa (AN) and assess its relationship with socio-emotional and academic adjustment following Leventhal’s Self-Regulation Model. Thirty-four female AN patients, with a mean age of 15.76 (SD = 2.00), completed the Revised Illness Perception Questionnaire (IPQ-R), the Psychosocial Adjustment to Illness Scale (PAIS) and the Hospital Anxiety and Depression Scale (HAD). Adolescent anorexia patients perceived that they had control over their illness and treatment would improve their condition. However, they also believed that it was highly distressing, going to last a long time and would have serious consequences. As for the causes of the disorder, they were attributed primarily to a specific eating disorder and psychological factors. The results showed that anorexia patients’ illness perceptions were related to socio-emotional and academic adjustment. In this sense, identity with the illness was associated with a worse academic adjustment, while emotional representation was associated with a worse emotional and social adjustment. These findings highlight how important it is for patients’ beliefs about their disease to be considered in the treatment of anorexia. In this respect, clinicians treating these patients should consider how certain beliefs affect their academic and socio-emotional adjustment. It would be interesting for patients to become aware of how these beliefs influence the strategies they use to cope with their disease as well as their adjustment to it.

## Introduction

Anorexia nervosa (AN) is a chronic and complex mental illness with serious physical and social consequences (Patel et al., 2016). Its prevalence ranges between 4 and 6% for women and under 1% in the case of men (DSM-5, American Psychiatric Association, 2013; Ward et al., 2019). It is characterized by a high resistance to treatment with serious family and social repercussions, including the highest mortality rate of any psychiatric illness (López and Treasure, 2011; Ward et al., 2019). Young women make up the majority of people with AN, with an average illness duration of about 6 years (Schmidt et al., 2016).

Anorexia nervosa is diagnostically defined by abnormal eating behavior and distorted attitudes toward body weight and shape (DSM-5). It has a high impact on patients’ quality of life, affecting their physical and psychological health as well as having important consequences for their academic and social functioning (Treasure et al., 2015).

Data suggest that AN is associated with atypical social and emotional functioning (Harrison et al., 2010; Patel et al., 2016). In this sense, negative social experiences (such as derogatory comments, negative feedback, and pressure) can be stressors that trigger the disease (Hutchinson and Rapee, 2007; Quiles et al., 2013), and during its most acute phase, patients can experience isolation and loneliness (Levine, 2012). It is also known that socio-communicative difficulties are maintaining factors for these patients (Patel et al., 2016; Schmidt and Treasure, 2006). Recently, Cardi et al. (2018) identified three specific factors that might be involved in the onset and maintenance of AN: fear of negative evaluation, perceived lack of social competence and early experiences of submissiveness.

Academic performance is another important trigger in AN patients. Although reported premorbid characteristics for these patients include high levels of academic performance and extreme perfectionism, when the illness becomes entrenched, cognitive decline and impaired concentration can accompany starvation and depression (Maxwell et al., 2011). This impaired neurocognitive functioning present in AN patients (Weider et al., 2015; Kucharska et al., 2019) leads to difficulties in different neuropsychological capacities, such as attention, working memory, long-term learning, autobiographical memory, processing speed, problem solving, or information processing (Oltra et al., 2012). Data suggests that this could be a consequence of the illness per se or could arise from intensified underlying traits for AN (Lang et al., 2016). On the one hand, the personal self-demand that characterizes these patients is associated with high pressure to obtain excellent academic results; and on the other hand, if patients identify themselves as “AN patients” their performance also worsens. However, academic performance can be affected by school stress too (Espíndola and Blay, 2009).

Cognitive difficulties are not the only factor that could influence academic performance (Yanover and Thompson, 2008), discrepancies between actual and ideal self-perceptions, what the patient would really like to be and how they really feel, are also a core symptom. These differences are related to poor school grades, low self-esteem and depressive symptoms (Ferguson et al., 2010). In fact, individuals with AN report that the social and psychological consequences of the disorder are more important than physical ones (Dovydaitiene and Maslauskiene, 2013). Thus, during the recovery process, it is important to consider the extent to which patients consider that the disease hinders their daily functioning (Tchanturia et al., 2012).

There is scarce research on illness perception with respect to AN (Jansen, 2016). It is perceived as a chronic illness with strong negative consequences (Holliday et al., 2005), and AN patients typically have a high number of symptoms that are difficult to control, cure, and understand. These perceptions are very important because patients with a strong belief in treatment control are more likely to report a better psychological adjustment and appear to have better academic performance, family relationships, and global adjustment (Quiles et al., 2007a). It is therefore apparent that illness perception is related to treatment outcomes, which can interfere with recovery. Moreover, AN is often seen as linked to an individual’s identity and difficult to give up (Higbed and Fox, 2010), and patients tend to perceive their illness as chronic and difficult to be treated. This perception of chronicity and severity is related to the impression that the eating disorder is untreatable (Stockford et al., 2007). Apart from this, illness perception also affects family and social relationships.

The aim of this study was to describe illness perception in adolescent AN patients and assess its relationship with socio-emotional and academic adjustment following Leventhal’s Self-Regulation Model (Leventhal et al., 1984, 1997, 2003). This is a very useful model to understand the way that AN patients understand and cope to the illness. It hypothesizes that individuals create mental representations of illness based on five dimensions (illness identity, timeline, consequences, causes, and controllability/curability).

## Materials and Methods

### Participants

A total of 34 female AN nervosa outpatients attending a specialized unit for eating disorders were recruited. Patients have a primary DSM-IV diagnosis of AN (restrictive type = 30; purging type = 4). Participants’ age ranged from 12 to 19 (Mean = 15.76 years, SD = 2.00). The patients had a mean illness duration of 25.82 months (SD = 18.62), and they were receiving psychological and nutritional treatment.

### Variables and Instruments

#### Sociodemographic and Clinical Characteristics

Patients’ sociodemographic data and clinical characteristics were collected through the completion of an *ad hoc* questionnaire. This sought information concerning ages and relevant clinical data for the study (diagnosis and history of the problem).

### Illness Perception

Patients’ perceptions of eating disorder were assessed using the specific eating disorders dimension from the Spanish version (Quiles et al., 2007b) of the Revised Illness Perception Questionnaire (IPQ-R; Moss-Morris et al., 2002). This questionnaire measures participants’ illness beliefs in eight dimensions: identity, timeline (individual’s beliefs about the course of the illness), consequences, cause, cyclical timeline, control, cure, and emotional representations. It is divided into three sections: the identity and causal dimensions are presented in two separate sections, while the remaining dimensions are presented together in one section. The cause subscale consists of four components: *psychological cause*, *ED specified cause*, *risk cause*, and *external cause*. The internal consistency for the subscales in the Spanish version of this questionnaire range from α = 0.55 to α = 0.85. For this study, McDonald’s omega for the subscales ranged from ω = 0.62 (cyclical timeline subscale) to ω = 0.86 (identity subscale).

#### Psychosocial Adaptation

The PAIS (Psychosocial Adjustment to Illness Scale, Derogatis, 1986) was used to assess adjustment to illness. The PAIS comprises 45 items, each of which contains four statements scored on a 4-point scale ranging from 0 (no problem) to 3 (many difficulties). Higher scores indicate more difficulties. The scale is designed to provide information on global adjustment as well as adjustment in specific areas. In this study, we have assessed these areas: educational and social adjustment, family relationships and psychological distress. The Spanish version of this questionnaire has adequate psychometric properties (from α = 0.70 to α = 0.90) (Neipp, 2005; Portillo et al., 2019). For this study, McDonald’s omega ranged from ω = 0.74 (educational adjustment) to ω = 0.91 (social adjustment).

#### Anxiety and Depression

The HAD (Hospital Anxiety and Depression Scale; Zigmond and Snaith, 1983) is a 14-item scale that assesses anxiety and depression, requiring answers on a 4-point scale. In this questionnaire, patients rate the degree to which they have experienced these symptoms over the previous week. The internal consistency, calculated with the Cronbach’s alpha coefficient for the Spanish version was 0.80 in the anxiety subscale and 0.76 in the depression subscale (Terol et al., 2007). For this study, McDonald’s omega for the subscales was ω = 0.73 and *ω* = 0.86, respectively.

#### Procedure

Patients were recruited from the Eating Disorder Unit of a public Hospital in the province of Alicante (Spain). Patients were included in the study if they were outpatients. Exclusion criteria were: borderline personality disorder, psychosis, current alcohol, substance dependence, or serious medical condition. This information was provided by the Eating Disorder Unit. These patients were handed information about the study during their treatment sessions. All patients and their relatives gave written informed consent for participation. Patients were interviewed in the Eating Disorder Unit by a qualified health psychologist after one of their treatment sessions.

### Data Analysis

A cross-sectional design was used for this study. Data were analyzed using IBM SPSS v.25. to obtain frequencies and data descriptions for mean and minimum and maximum standard deviation. A one-way ANOVA was carried out to determine the differences in illness perception scores according to illness duration. For relation analysis, we used the Pearson correlation coefficient, and a stepwise multiple regression procedure was conducted to examine the relation between illness perception and socio-emotional and academic adjustment. Prior to the analysis, the assumptions of normality, homoscedasticity and independence were tested. The three assumptions were met.

## Results

### Illness Perception

#### Identity Subscale

Illness identity refers to statements regarding beliefs about the illness label and the link with symptoms. The mean for symptoms experienced by the patients was 6.85 (SD = 2.94). The two most frequently experienced symptoms were “weight loss” (94.1%) and “irregularities in menstruation” (88.2%). The two least frequently experienced symptoms were “wheeziness,” “sore eyes” and “stiff joints.” Results for this subscale are given in Table 1.

**TABLE 1:** Identity subscale: frequency of experienced symptoms.

	***N***	**%**
1. Pain	19	55.9
2. Sore throat	11	32.4
3. Nausea	12	35.3
4. Breathlessness	9	26.5
5. Dry and rough skin	26	76.5
6. Irregularities in menstruation	30	88.2
7. Weight loss	32	94.1
8. Fatigue	20	58.8
9. Stiff joints	7	20.6
10. Sore eyes	7	20.6
11. Wheeziness	3	8.8
12. Headache	17	50
13. Upset stomach	27	79.4
14. Sleep difficulties	15	44.1
15. Dizziness	18	52.9
16. Loss of strength	23	67.6

#### Consequences, Control, Cure, Timeline, Emotional Representation and Cyclical Timeline Subscales

Illness perception of the sample is described in Table 2. The mean scores for these dimensions would suggest that these patients’ perception of their illness is that it was highly distressing, they had control over it, it was going to last a long time, and it had serious consequences. A one-way ANOVA comparing illness perception according to illness duration revealed that AN patients with a shorter average illness duration (≤1 year), perceived their illness with less serious consequences (*M* = 10; SD = 3.36) than patients with a longer illness duration (≥2 years) (*M* = 12.5; SD = 1.67) (*F* = 4.06, *p* < 0.05).

**TABLE 2:** Means and standard deviations of illness perception dimensions.

**Dimension**	**Range/mean point**	***M***	**SD**
Timeline	3–15 (9)	16.58	6
Control	5–25 (15)	24.85	3.08
Cure	5–25 (15)	19.94	3.08
Consequences	3–15 (9)	10.88	2.66
Cyclical timeline	3–15 (9)	9	2.94
Emotional representation	5–25 (15)	22.7	5.44

#### Causal Subscale

These patients showed higher scores in “specified ED factor” (*M* = 23.52; SD = 6.35) and “psychological factor” (*M* = 20.26; SD = 6.19). By contrast, the lowest scores were in “external cause factor” (*M* = 6; SD = 2.65) and in “risk factor” (*M* = 12.61; SD = 4). The factors that were most frequently identified as the cause of anorexia were low self-esteem and own behavior, followed by the need to be perfect, while the least frequently identified factors were alcohol, germen or virus and smoking. A one-way ANOVA comparing the type of causes to which they attributed their illness according to illness duration revealed that AN patients with the shortest average illness duration (≤6 months), attributed their disease to causes of the specific ED factor to a lesser extent (*M* = 18.00; SD = 6.00) than the group with the longest evolution time (≥4 years) (*M* = 28.8; SD = 5.8) (*F* = 3.85, *p* < 0.05).

### Relationships Between Illness Perception and Socio-Emotional and Academic Adjustment

The Pearson correlation analyses showed that “identity” was associated with psychological distress (*r* = 0.48, *p* < 0.01), academic adjustment (*r* = 0.51, *p* < 0.01), family relationships (*r* = 0.45, *p* < 0.01), anxiety (*r* = 0.40, *p* < 0.05), and depression (*r* = 0.36, *p* < 0.05). Emotional representation was associated with psychological distress (*r* = 0.68, *p* < 0.001), anxiety (*r* = 0.50, *p* < 0.01), depression (*r* = 0.54, *p* < 0.01), family relationships (*r* = 0.43, *p* < 0.05), and with social (*r* = 0.36, *p* < 0.05) and academic (*r* = 0.38, *p* < 0.05) adjustment.

The timeline dimension was associated with psychological distress (*r* = 0.42, *p* < 0.01) and anxiety (*r* = 0.36, *p* < 0.05). ED specified causes were associated with family relationships (*r* = 0.38, *p* < 0.05) and with anxiety (*r* = 0.39, *p* < 0.05).

The results of the regression analyses are shown in Table 3.

**TABLE 3:** Stepwise multiple regression analyses: the relation between illness perception and socio-emotional and academic adjustment.

**Dependent variables/predictors**	***R*^2^*/r***	**Change *R*^2^**	***F***	**β**
**Emotional adjustment**				
Anxiety/emotional representation	0.25/0.5	0.25	10.96*	0.50*
Depression/emotional representation	0.29/0.53	0.29	13.17**	0.54**
Psychological distress/emotional representation	0.50/0.70	0.47	18.06***	0.68***
Personal control		0.06		−0.25*
**Academic and social adjustment**				
Academic adjustment/identity	0.24/0.48	0.26	11.42*	0.51*
Family relationships/identity	0.18/0.42	0.20	8.38*	0.45**
Social adjustment/emotional representation	0.10/0.31	0.12	4.70*	0.36*

*p < 0.05, **p < 0.01, ***p < 0.001.

“Emotional representation” was the most significant illness perception dimension related to emotional adjustment. It contributed to 25% of the variance in anxiety, 29% in depression, and 47% in psychological distress.

With respect to academic adjustment, “identity” contributed to 24% of the variance, while “emotional representation” explained 10% of the variance, which was the same as in social adjustment.

## Discussion

Illness perception in adolescent AN patients may not coincide with the clinical conception of this disorder, and their understanding of this illness and its causes, consequences, duration, and treatment effectiveness may significantly influence their recovery process. The results of this study showed that adolescent AN patients reported experiencing a moderate number of physical symptoms and believed that they had control over their disease and that the treatment was effective for their recovery. However, they also believed that their illness was going to last a long time and that it had serious consequences. The most commonly reported causes were attributed to the “specified ED factor” (refers to items that have been identified in research as core causes related to the onset of EDs) and “psychological factor,” such as low self-esteem and the need to be perfect. AN patients with longer duration of the illness perceived more physical and psychological complications.

This research has shown that AN patients’ illness perceptions are related to socio-emotional and academic adjustment. One area of concern is the extent to which negative emotional responses to AN negatively influence patients’ socio-emotional adjustment. These results are in line with Leventhal’s Self-Regulation Model, which asserts that emotional representation is a determining factor in an individual’s emotional response to their disease (Leventhal et al., 1980). In this context, high levels of illness-related distress (emotional representation) are associated with anxiety, depression, psychological distress and worse social functioning. Thus, these unpleasant emotions will affect patients’ social adjustment and therefore deteriorate their social relations. If an individual is afraid and insecure or perceives any situation as a threat or danger, it is difficult for them to relate to others, and consequently, these feelings can lead to social isolation, which is a key maintaining factor of the disorder (Kan and Treasure, 2019; Meneguzzo et al., 2020).

Illness identity or the tendency to attribute symptoms to AN was associated with academic adjustment, since these symptoms can become a signal to patients that their AN is active or progressing, leading to anxiety (Leventhal et al., 2003). Literature has shown that high levels of anxiety reduce learning efficiency by decreasing attention, concentration and retention, with the consequent deterioration in school performance (Van Ameringen et al., 2003; Visu-Petra et al., 2014; Respondek et al., 2017). For this reason, it is very important to treat anxiety that comes from identifying symptoms because decreased school performance negatively affects marks as well as adolescent students’ self-esteem (Nguyen et al., 2019).

This study highlights that illness perception is a key factor to be considered and one that should be included in the treatment of anorexia. In this sense, interventions designed to address illness perception can improve adjustment in these patients. Despite the importance of patients’ beliefs as well as their views of their symptoms and illness, they are not fully discussed in consultations. The results of the psychological treatment of AN may improve if the therapist addresses patients’ perceptions of identity and the emotional state of these patients.

Clinicians supporting adolescent AN patients’ adjustment should consider that some illness beliefs are associated with their academic and socio-emotional adjustment. It would be interesting for these patients to become aware of how these beliefs influence the strategies they use to cope with their disease as well as their adjustment to it.

This research has produced clear findings, but a number of limitations need to be considered, as sample size and the low reliability of some subscales, as cyclical timeline subscale of the IPQ-R. The results of the study might be influenced by the fact that at the time of the study all patients were in outpatient treatment. Therefore, it is likely that the results cannot be generalized to other patient samples. This was a cross-sectional study, so it would be important to carry out a longitudinal study to find out how illness perception changes in response to experience with one’s illness.

## Data Availability Statement

The raw data supporting the conclusions of this article will be made available by the authors, without undue reservation.

## Ethics Statement

The studies involving human participants were reviewed and approved by University Miguel Hernández. Written informed consent to participate in this study was provided by the participants’ legal guardian/next of kin.

## Author Contributions

YQ, MQ, and EL developed the design of the research. YQ and JM performed the data analyses. MQ, EL, and JM wrote the first draft. All authors contributed and reviewed the final draft. All authors participated in the development of the study and approved the manuscript.

## Conflict of Interest

The authors declare that the research was conducted in the absence of any commercial or financial relationships that could be construed as a potential conflict of interest.
